# Coenzyme Q10 Inhibits the Aging of Mesenchymal Stem Cells Induced by D-Galactose through Akt/mTOR Signaling

**DOI:** 10.1155/2015/867293

**Published:** 2015-02-18

**Authors:** Dayong Zhang, Bingxi Yan, Shanshan Yu, Chong Zhang, Baoming Wang, Yayan Wang, Junbo Wang, Zhanggen Yuan, Lihuang Zhang, Jianping Pan

**Affiliations:** Department of Clinical Medicine, Zhejiang University City College School of Medicine, Hangzhou 310015, China

## Abstract

Increasing evidences indicate that reactive oxygen species are the main factor promoting stem cell aging. Recent studies have demonstrated that coenzyme Q10 (CoQ10) plays a positive role in organ and cellular aging. However, the potential for CoQ10 to protect stem cell aging has not been fully evaluated, and the mechanisms of cell senescence inhibited by CoQ10 are still poorly understood. Our previous study had indicated that D-galactose (D-gal) can remarkably induce mesenchymal stem cell (MSC) aging through promoting intracellular ROS generation. In this study, we showed that CoQ10 could significantly inhibit MSC aging induced by D-gal. Moreover, in the CoQ10 group, the expression of p-Akt and p-mTOR was clearly reduced compared with that in the D-gal group. However, after Akt activating by CA-Akt plasmid, the senescence-cell number in the CoQ10 group was significantly higher than that in the control group. These results indicated that CoQ10 could inhibit D-gal-induced MSC aging through the Akt/mTOR signaling.

## 1. Introduction

Adult stem cells are critical for organ-specific regeneration and self-renewal with advancing age [1]. Mesenchymal stem cells (MSCs) have emerged as a reliable cell source for stem cell transplantation and are currently being tested in numerous ongoing clinical trials [2, 3]. Unfortunately, recent studies have shown that MSC function declines in older individuals and that MSC dysfunction influences the effects of autologous MSC transplantation in older individuals [4–6]; our previous study also showed that MSC aging could be induced by old rat serum [7]. Therefore, it is significant to find a useful method to delay MSC senescence.

The balance between prooxidants and antioxidants is critical for survival and function of MSCs [8]. Increasing evidences showed that intracellular reactive oxygen species (ROS) is the main factor promoting stem cell aging [9–11]. Our previous study showed that ROS were the main mediators of MSC aging induced by excessive activation of Wnt/*β*-catenin signaling [12]. Therefore, it is important to find out effective and safe antioxidants that inhibit the oxidative damage and cellular aging induced by ROS.

Coenzyme Q10 (CoQ10) or ubiquinone (2,3-dimethoxy-5-methyl-6-polyprenyl-1,4-benzoquinone) is a lipophilic molecule found in the phospholipid bilayer of cellular membranes and is present in especially high concentration in the mitochondrial inner membrane [13]. In vivo supplementation of CoQ10 to rats fed a high-fat diet reduced DNA double stranded breaks in peripheral blood mononuclear cells and increased lifespan [14]. CoQ10 is widely available as a dietary supplement and remains under consideration as a treatment for age-associated neurodegenerative conditions [15, 16]. As a component of mitochondrial electron transport chain, CoQ10 can increase mitochondrial mass [17], improve mitochondrial function [18], and inhibit ROS generation [19]. The importance of CoQ10 in mitochondrial function [20, 21] and its status as an antioxidant have led to therapeutic applications and clinical trials in neurodegenerative diseases [15, 22, 23]; however, the potential for CoQ10 to ameliorate or reverse stem cell aging has not been fully evaluated. In current study, D-galactose (D-gal), a ROS promoter [24], was used to induce MSC aging; we explored the protecting effects of CoQ10 on MSC aging induced by D-gal.

Studies have demonstrated that CoQ10 has antioxidative effects and antiaging properties at the skin level [25] and cardiac tissue [26] and in spatial learning [27]. However, the molecular mechanisms of CoQ10 inhibiting cell aging are still poorly understood. Recent studies also suggest that phosphatidylinositol 3-kinase (PI3K)/Akt and mammalian target of rapamycin (mTOR) are associated with stem cell aging [28–30]. Interestingly, links are emerging between Akt/mTOR signaling and ROS with evidence suggesting reciprocal regulation of these pathways by each other [31, 32]. For instance, high levels of mTOR have been shown to correlate with increase in ROS generation [33, 34]. Therefore, in the present study, we explored whether CoQ10 can inhibit MSC aging through Akt/mTOR pathway.

Although CoQ10 has been found to have antiaging effects on rats, the effect and molecular mechanisms by which CoQ10 modulates ROS generation and stem cell aging are still unclear. In this paper, we demonstrate, for the first time, that CoQ10 can attenuate MSC aging by inhibiting ROS generation, and the Akt/mTOR signaling may play a critical role in MSC aging inhibited by CoQ10.

## 2. Materials and Methods

### 2.1. Isolation and Culture of MSCs

Seven-day-old Sprague-Dawley (SD) rats were obtained from Zhejiang Medical Academy of Science (Permit number SCXK (Zhejiang) 2008-0033). The investigation was permitted by the Law of the People's Republic of China on the Protection of Wildlife, and the protocol was approved by Zhejiang Medical Academy of Science, China. The femurs and tibias were removed from the SD rats and bone marrow was flushed out of the bones using 10 mL PBS with 100 U/mL heparin in a syringe. The cells were centrifuged at 1,000 rpm for 8 min. The cell pellet was resuspended in 2.5 mL Dulbecco's modified Eagle's medium (DMEM, Gibco, USA) supplemented with 15% fetal bovine serum (FBS, Gibco, USA) and plated in a 25 cm^2^ plastic flask (Corning, USA) to allow the MSCs to adhere. After 3 days, the medium was changed and the nonadherent cells were discarded. The medium was completely replaced every 3 days. The adherent cells were released from the dishes with 0.25% trypsin (Gibco, USA) and seeded into new fresh culture flasks. All the experiments described below were performed using MSCs from the third to the fifth passages.

### 2.2. Plasmids and Transfections

CA-Akt plasmid, a constitutively active form of mouse Akt-1, was friendly provided by Michael Robinson (Pennsylvania University). CA-Akt or a control plasmid (pCMV) transfection was performed using Lipofectamine 2000 (Invitrogen, USA) using 1 *μ*g plasmid per reaction according to the protocol; cells were left to incubate for 12 h before harvest or drug treatment.

### 2.3. Treatment of MSCs

MSCs were divided into the following four groups. (1) Control group: MSCs were cultured for 48 h in DMEM containing 10% FBS. (2) D-gal treatment group: MSCs were incubated for 48 h in DMEM containing 10% FBS in the presence of 1, 10, or 100 g/L D-gal (Sigma, USA), respectively. (3) CoQ10 treatment group: CoQ10 (Sigma, USA) was dissolved in dimethyl sulfoxide (DMSO, Sigma, USA) as a stock solution at concentrations of 1, 10, and 100 mmol/L, respectively, immediately before use, which was diluted further 1000-fold in DMEM containing 10% FBS. The cells were incubated in the culture medium containing 1, 10, or 100 *μ*mol/L CoQ10 and 10 g/L D-gal for 48 h. The same volume of DMSO (0.1% v/v) was added to D-gal control group that contained only 10 g/L D-gal. (4) CA-Akt group: the cells were first transfected with CA-Akt plasmid for 12 h; then 10 g/L D-gal and 10 *μ*mol/L CoQ10 were added to the culture medium and cells were incubated further for 48 h.

### 2.4. SA-*β*-Gal Staining

Senescence-associated *β*-galactosidase (SA-*β*-gal) staining was performed using a SA-*β*-gal staining kit (Beyotime, China) following the manufacturer's protocol. The cells were fixed in 4% (v/v) formaldehyde for 5 min and then were stained with SA-*β*-gal staining solution at pH6.0 for 12 h. The SA-*β*-gal-positive cells exhibited a blue color. The number of positive cells was counted under a phase-contrast microscope. The experiment was repeated five times in each group.

### 2.5. Determination of Intracellular and Mitochondrial ROS Levels

Intracellular or mitochondrial ROS staining was performed using an DCFH-DA staining kit (Genmed, USA) or MitoSOX Red (Invitrogen, USA) following the manufacturer's protocol. The cells treated with 100 *μ*mol/L H_2_O_2_ for 60 min served as a positive control. After being cultured in each group, according to the above treatment methods, the cells were washed three times in PBS and incubated in DCFH-DA (30 *μ*mol/L) or MitoSOX Red (5 *μ*mol/L) solution at 37°C for 20 min. After washing, the nuclei were counterstained with Hoechst 33342 (Sigma, USA). The cells were observed using a fluorescence microscope (Eclipse 50I, Nikon, Japan). To quantify the ROS level, the DCFH or MitoSOX Red fluorescence intensity was detected by flow cytometer (Calibur, BD Biosciences, USA) at excitation/emission maxima of 488/525 nm or 488/580 nm. Experiments were repeated five times.

### 2.6. Western Blot Analysis

To assay the expression of p16^INK4a^, p53, p21, p-Akt, and p-mTOR, the total cellular protein was extracted through the following methods: MSCs from different treatment groups were washed in cold-buffered PBS and were then lysed in RIPA buffer (150 mM NaCl, 1% Triton X-100, 0.5% NaDOD, 0.1% SDS, and 50 mM Tris, pH 8.0). After centrifugation (12,000 rpm, 5 min) at 4°C, the protein supernate was transferred into new tubes. The protein concentration of the samples was determined with a bicinchoninic acid protein assay (Beyotime, China). A 40 *μ*g sample of the total protein was resolved using 12% SDS-PAGE and transferred onto polyvinylidene difluoride (PVDF, Millipore, USA) membranes. The membranes were blocked with 5% nonfat milk at room temperature for 1 h in Tris-buffered saline containing Tween 20 (TBST). Primary antibodies to detect p16^INK4a^ (1 : 1000, Santa Cruz, USA), p53 (1 : 2000, BD, USA) and p21 (1 : 1000, Santa Cruz, USA), p-Akt (Ser473, 1 : 2000, CST, USA), p-mTOR (Ser2448, 1 : 2000, CST, USA), and *β*-actin (1 : 5000, BD, USA) were incubated overnight with the membranes at 4°C. Membranes were incubated with horseradish peroxidase- (HRP-) conjugated anti-rabbit or anti-mouse secondary antibodies (1 : 2000, Dako, USA), and proteins were detected by enhanced chemiluminescence (ECL) (Amersham Biosciences Corp., USA). *β*-actin was used as the internal control to normalize the loading materials.

### 2.7. Statistical Analysis

All experiments were performed at least in triplicate. All data are presented as mean ± standard deviation of the mean (SD). Significance testing was performed using one-way analysis of variance (ANOVA) to compare data from different experimental groups. A level of *P* < 0.05 was considered as statistically significant.

## 3. Results

### 3.1. D-gal Promotes MSCs Senescence

**Figure 1:**
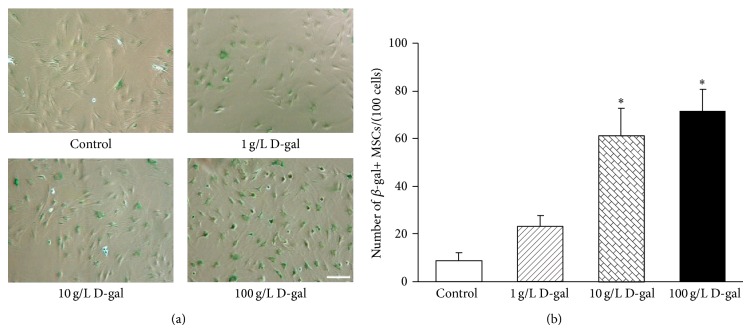
Effects of different concentrations D-gal on MSCs senescence. (a) SA-*β*-gal staining. Compared with the control group, the number of SA-*β*-gal-positive cells in the 10 g/L D-gal and the 100 g/L D-gal group was clearly increased, and those SA-*β*-gal-positive cells show flat and enlarged cell shape. Scale bar = 25 *μ*m. (b) Quantification of SA-*β*-gal-positive cells. The total number of SA-*β*-gal-positive cells among 100 random cells was counted using phase-contrast microscopy. The results showed that the number of SA-*β*-gal-positive MSCs/100 cells in the 10 g/L D-gal and the 100 g/L D-gal groups was significantly higher than that in the control group (^*^ *P* < 0.01; *n* = 5).

To explore the effects of D-gal on MSCs senescence, we examined cellular senescence through SA-*β*-gal staining. As shown in Figure 1(a), only several SA-*β*-gal-positive cells were seen in the control group. However, in the 10 g/L D-gal and the 100 g/L D-gal group, the number of SA-*β*-gal-positive cells was clearly increased, and those SA-*β*-gal-positive cells showed flat and enlarged cell shape. The cell count revealed that the number of SA-*β*-gal-positive cells was significantly increased in the 10 g/L D-gal (61.4 ± 11.3/100 cells) and the 100 g/L D-gal groups (71.4 ± 9.1/100 cells) than that in the control group (8.8 ± 3.3/100 cells, *P* < 0.01, Figure 1(b)). The results indicated that 10 g/L or 100 g/L D-gal could clearly promote MSCs senescence. So we used 10 g/L D-gal to induce MSCs senescence in follow-up experiment, which was named the D-gal control group.

### 3.2. CoQ10 Attenuates D-gal-Induced MSCs Senescence

**Figure 2:**
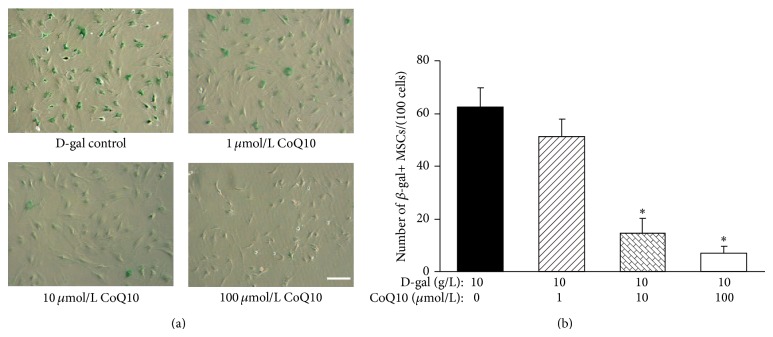
Effects of different concentrations CoQ10 on MSCs senescence induced by D-gal. (a) SA-*β*-gal staining. In the 10 *μ*mol/L CoQ10 and the 100 *μ*mol/L CoQ10 groups, the number of SA-*β*-gal-positive cells was clearly decreased compared with that in the D-gal control group. Scale bar = 25 *μ*m. (b) Quantification of SA-*β*-gal-positive cells. The total number of SA-*β*-gal-positive cells among 100 random cells was counted using phase-contrast microscopy. The results showed that the number of SA-*β*-gal-positive MSCs/100 cells in the 10 *μ*mol/L CoQ10 and the 100 *μ*mol/L CoQ10 groups was significantly lower than that in the D-gal control group (^*^ *P* < 0.01; *n* = 5).

After incubation with different concentrations of CoQ10 for 48 h, the influence of CoQ10 on MSC senescence induced by D-gal was examined. As shown in Figure 2(a), with the increase of CoQ10 concentration, the number of SA-*β*-gal-positive cells in 1, 10, and 100 *μ*mol/L CoQ10 groups was gradually decreased. Particularly, there were only a few SA-*β*-gal-positive cells that could be seen in the 100 *μ*mol/L CoQ10 group. Quantification of SA-*β*-gal-positive cells showed that the number of SA-*β*-gal-positive cells in the 10 and 100 *μ*mol/L CoQ10 groups (14.6 ± 5.6 and 6.8 ± 2.9/100 cells) was significantly decreased compared with that in the D-gal control group (62.6 ± 7.1/100 cells, *P* < 0.01, Figure 2(b)). The results suggested that D-gal-induced MSCs aging could be significantly attenuated by CoQ10.

### 3.3. CoQ10 Inhibits D-gal-Induced Intracellular and Mitochondrial ROS Generation in MSCs

**Figure 3:**
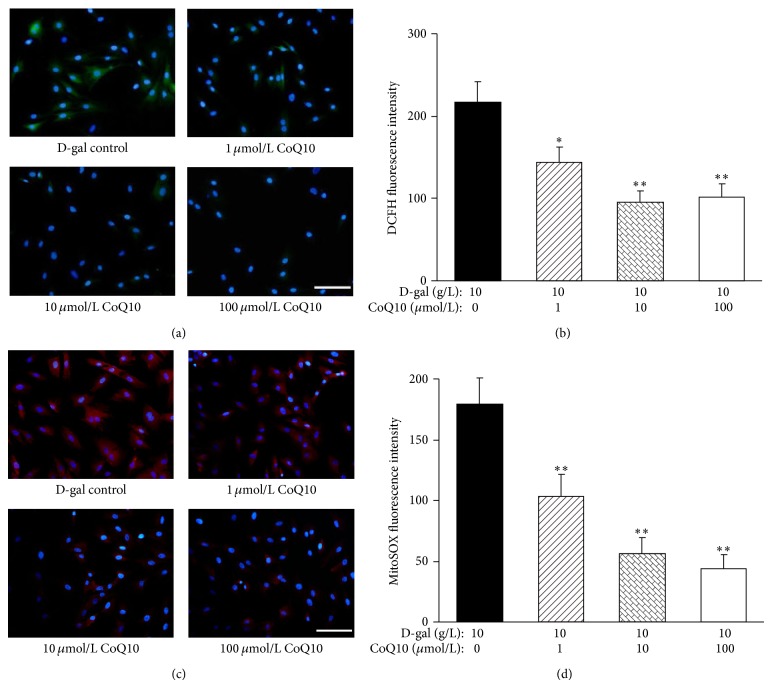
Effects of different concentrations CoQ10 on ROS generation in MSCs. (a) DCFH staining. In the 1, 10, and 100 *μ*mol/L CoQ10 group, little DCFH-stained cells were observed under a fluorescence microscope compared with that in the D-gal control group. Green: DCFH staining; blue: Hoechst 33342 staining. Scale bar = 25 *μ*m. (b) Quantification of intracellular ROS level. The DCFH fluorescence intensity in the 1, 10, and 100 *μ*mol/L CoQ10 group was evidently lower compared with the D-gal control group (^*^ *P* < 0.05, ^**^ *P* < 0.01; *n* = 5). (c) MitoSOX staining. In the 1, 10, and 100 *μ*mol/L CoQ10 group, MitoSOX fluorescence brightness was clearly lower than that in the D-gal control group. Red: MitoSOX staining; blue: Hoechst 33342 staining. Scale bar = 25 *μ*m. (d) Quantification of mitochondrial ROS level. The MitoSOX fluorescence intensity in the 1, 10, and 100 *μ*mol/L CoQ10 group was significantly lower compared with the control group (^**^ *P* < 0.01; *n* = 5).

DCFH staining showed that the number of ROS-stained cells and the DCFH fluorescent level of the cells were gradually decreased as the increase of the concentration of CoQ10 (Figure 3(a)). Quantification of the DCFH fluorescence intensity showed that the intensity of DCFH fluorescence in the 10 and 100 *μ*mol/L CoQ10 groups (96.0 ± 12.7 and 102.0 ± 15.9) was significantly decreased compared with that in the D-gal control group (216.6 ± 25.2, *P* < 0.01; Figure 3(b)). In order to determine the effect of CoQ10 on mitochondrial ROS generation, a fluorescent probe, MitoSOX Red, was used as a specific marker for quantitative mitochondrial ROS accumulation. Compared to the D-gal control group, the MitoSOX fluorescence intensity in the CoQ10 treatment group was significantly decreased (*P* < 0.01; Figures 3(c) and 3(d)). These results indicated that CoQ10 could clearly extenuate the promoting effect of D-gal on intracellular and mitochondrial ROS production in MSCs, which may be the main mechanism that CoQ10 can delay MSCs senescence induced by D-gal.

### 3.4. CoQ10 Inhibits the Expression of p16^INK4a^, p53, and p21 in MSCs

**Figure 4:**
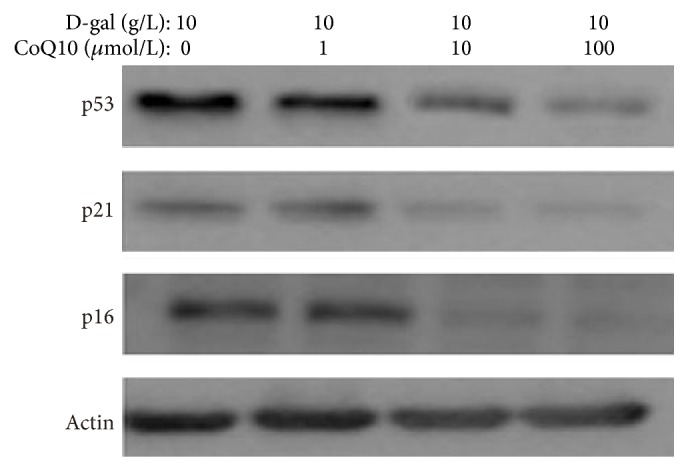
Effects of different concentrations CoQ10 on the expression of p16^INK4a^, p53, and p21 in MSCs. Protein expression of p16^INK4a^, p53, and p21 was examined by western blot. *β*-actin was used as the internal control. The p16^INK4a^, p53, and p21 expression levels were gradually lower in the 1, 10, and 100 *μ*mol/L CoQ10 group compared with those in the D-gal control group.

To investigate the effects of CoQ10 on the expression of senescence-related proteins, we examined p16^INK4a^, p53, and p21 expression by western blot analysis. The results showed that, in the 1, 10, and 100 *μ*mol/L CoQ 10 groups, the expression of p16^INK4a^, p53, and p21 was gradually decreased compared with that in the D-gal control group (Figure 4). The results hinted that p53/p21 and p16^INK4a^ might play mediating roles in MSCs senescence delayed by CoQ10.

### 3.5. CoQ10 Decreases the Expression of p-Akt and p-mTOR in MSCs

**Figure 5:**
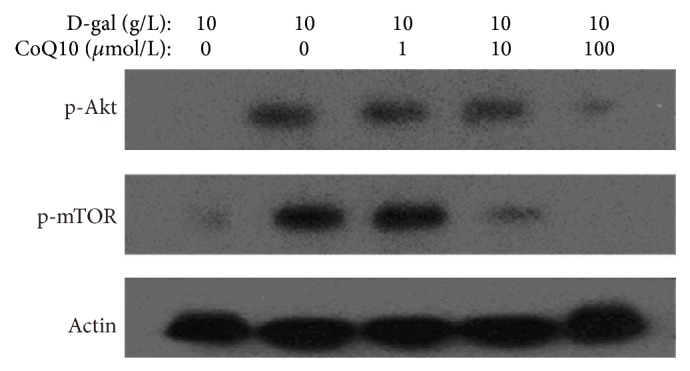
Effects of CoQ10 on the expression of p-Akt and p-mTOR in MSCs. Western blot analysis of p-Akt and p-mTOR protein expression. *β*-actin was used as the internal control. The p-Akt and p-mTOR expression levels were gradually lower in the 1, 10, and 100 *μ*mol/L CoQ 10 group compared with those in the D-gal control group.

To explore the effects of CoQ10 on the Akt/mTOR signaling in MSCs, we examined p-Akt and p-mTOR expression through western blot analysis in MSCs. The results showed that the expression of p-Akt and p-mTOR was clearly increased in the D-gal control group when compared with that in the negative control group. However, with the increase of the concentration of CoQ10, the expression of p-Akt and p-mTOR was gradually decreased in the 1, 10, and 100 *μ*mol/L CoQ10 groups compared with that in the D-gal group (Figure 5). The results suggested that CoQ10 could inhibit D-gal-activated Akt/mTOR signaling in MSCs.

### 3.6. Akt/mTOR Signaling Plays Key Role in MSC Aging Inhibited by CoQ10

**Figure 6:**
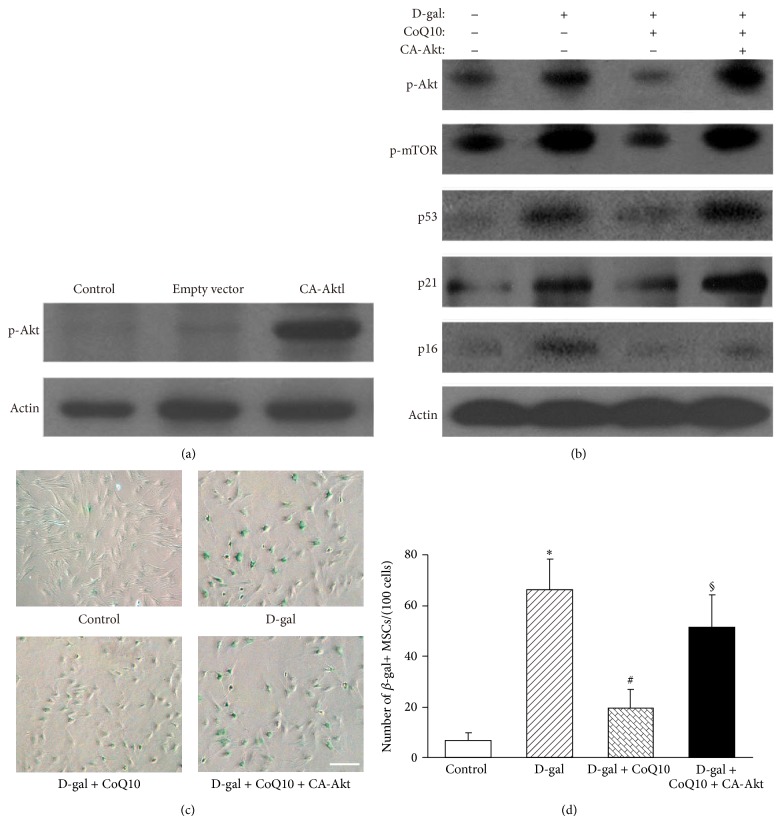
Effects of Akt/mTOR signaling on MSC aging inhibited by CoQ10. (a) Western blot analysis. The p-Akt expression level was evidently higher in the CA-Akt group compared with that in the control group or the empty vector group. *β*-actin was used as the internal control. (b) Western blot analysis. The p-Akt, p-mTOR, p16^INK4a^, p53, and p21 expression levels were higher in the D-gal group compared with those in the control group. After treatment with CoQ10 in MSCs for 48 h, the p-Akt, p-mTOR, p16^INK4a^, p53, and p21 expression levels were lower compared with those in the D-gal group. However, in the D-gal + CoQ10 + CA-Akt group, the p-Akt, p-mTOR, p16^INK4a^, p53, and p21 expression levels were increased. *β*-actin was used as the internal control. (c) SA-*β*-gal staining. Compared with the control group, the number of SA-*β*-gal-positive cells in the D-gal group was clearly increased. In the D-gal + CoQ10 group, the number of SA-*β*-gal-positive cells was obviously decreased compared with that in the D-gal group. However, after constitutive overexpression of Akt, the number of SA-*β*-gal-positive cells in the D-gal + CoQ10 + CA-Akt group was clearly increased compared with the D-gal + CoQ10 group. Scale bar = 25 *μ*m. (d) Quantification of SA-*β*-gal-positive cells. The total number of SA-b-gal-positive cells among 100 random cells was counted using phase-contrast microscopy. The results show that the number of SA-*β*-gal-positive MSCs/100 cells in the D-gal group was significantly higher than that in the control group (^*^ *P* < 0.01; *n* = 3). In the D-gal + CoQ10 group, the number of SA-*β*-gal-positive cells was obviously decreased compared with that in the D-gal group (^#^ *P* < 0.01; *n* = 3). In the D-gal + CoQ10 + CA-Akt group, the number of SA-*β*-gal-positive cells was clearly increased compared with the D-gal + CoQ10 group (^§^ *P* < 0.01; *n* = 3).

To further define the role of Akt/mTOR signaling in MSC senescence attenuated by CoQ10, we expressed constitutively active Akt isoforms in MSC, using a myristoylated constitutively active Akt (CA-Akt) plasmid, to test whether activation of Akt alone was sufficient to reverse the attenuating effect of CoQ10 on MSC senescence. Western blot analyses showed that the p-Akt expression level was evidently higher in the CA-Akt group compared with that in the control group and the empty vector group (Figure 6(a)). However, the effects of CoQ10 inhibition on p53, p21, and p16^INK4a^ expression were clearly reversed by CA-Akt; the expression of p53, p21, and p16^INK4a^ was higher in the D-gal + CoQ10 + CA-Akt group than that in the D-gal + CoQ10 group (Figure 6(b)). SA-*β*-gal staining showed that the number of SA-*β*-gal-positive cells in the D-gal group was clearly increased compared with the control group (66.2 ± 12.2/100 cells versus 6.6 ± 3.1/100 cells, *P* < 0.01). In the D-gal + CoQ10 group, the number of SA-*β*-gal-positive cells was obviously decreased than that in the D-gal group (19.4 ± 7.5/100 cells versus 66.2 ± 12.2/100 cells, *P* < 0.01). However, in the D-gal + CoQ10 + CA-Akt group, the number of SA-*β*-gal-positive cells was clearly increased compared with the D-gal + CoQ10 group (51.4 ± 12.7/100 cells versus 19.4 ± 7.5/100 cells, *P* < 0.01, Figures 6(c) and 6(d). These data indicate that the Akt/mTOR signaling plays a critical role in MSCs senescence inhibited by CoQ10.

## 4. Discussion

MSCs are characterized by their ability to self-renew and to differentiate into multiple cell lineages [35, 36] and have been widely used in clinical cell transplantation therapy [37, 38]. However, increasing studies have shown that aging of MSCs affects their clinical application [7, 39–41]. Reactive oxygen species are a major compartment that is known to regulate MSC senescence [12, 42, 43]. A major compartment or niche that is known to regulate quiescence and multipotency of MSC is the level of oxygen [44]. In fact, a hypoxic microenvironment is considered to be an important component of bone marrow, even at these sites ROS production increases with age and causes oxidative DNA damage resulting in reduced stem cell function [45, 46]. High oxygen tension and generation of ROS are well documented to be major factors in MSC aging and loss of stem cell properties [46–48]. D-gal-induced aging frequently was used as an experimental model for studying aging and to design suitable drug strategies against aging [49–51]. Our previous study had also showed that D-gal could significantly induce MSC aging through promoting ROS generation [52]. Herein, we also found that the number of SA-*β*-gal-positive MSCs was significantly increased in the 10 g/L and 100 g/L D-gal groups.

CoQ10 is a vitamin-like, oil-soluble molecule, and its reduced form is a potent lipophilic antioxidant. Studies have demonstrated that CoQ10 has antioxidative effects and antiaging properties at the skin level [25] and in spatial learning [27]. Several recent studies have shown that CoQ10 has an important protective role in astrocytes [53], human endothelial cells [54], hair cells [55], and neuronal cells [56]. However, it is still unclear whether CoQ10 offers delay benefits to stem cell aging. Here, we demonstrated that CoQ10 had clearly protecting effects on MSC aging induced by D-gal and could obviously decrease the level of intracellular and mitochondrial ROS in D-gal-treated MSCs. These results suggested that CoQ10 was an effective antioxidant on MSC and had obviously protective effects on MSC aging induced by ROS. Increasing studies have demonstrated that CoQ10 can reverse the mitochondrial dysfunction and decrease mitochondrial ROS generation [18, 57–59], which is consistent with the results of the present study. Therefore, it is possible that CoQ10 can also suppress dysfunction and aging of MSCs.

The current study addressed the main mechanism of the protective effects of CoQ10 on cellular aging was that could decrease the oxidative stress [26, 60]. However, ROS, as a kind of signaling factor, are related with some intracellular pathways [31, 61, 62]; many of these signaling pathways are involved in regulation of cell senescence [63]. The Akt/mTOR pathway plays a crucial role in stem cell aging [64, 65], and this signaling could be inhibited by ROS in cancer cells [66, 67]. Therefore, we hypothesized that Akt/mTOR signaling inactivation might be the mechanism by which CoQ10 inhibited MSC aging induced by D-gal. Our result showed that the expression of phosphorylated Akt and mTOR could be deceased by CoQ10. Finally, after overexpression of constitutively active Akt (CA-Akt), the number of SA-*β*-gal–positive cells was increased and the level of p53, p21, and p16 was also elevated in the CoQ10 treatment group; these results hint that the Akt/mTOR signaling may be the main mediator of MSC aging regulated by CoQ10.

## 5. Conclusion

**Figure 7:**
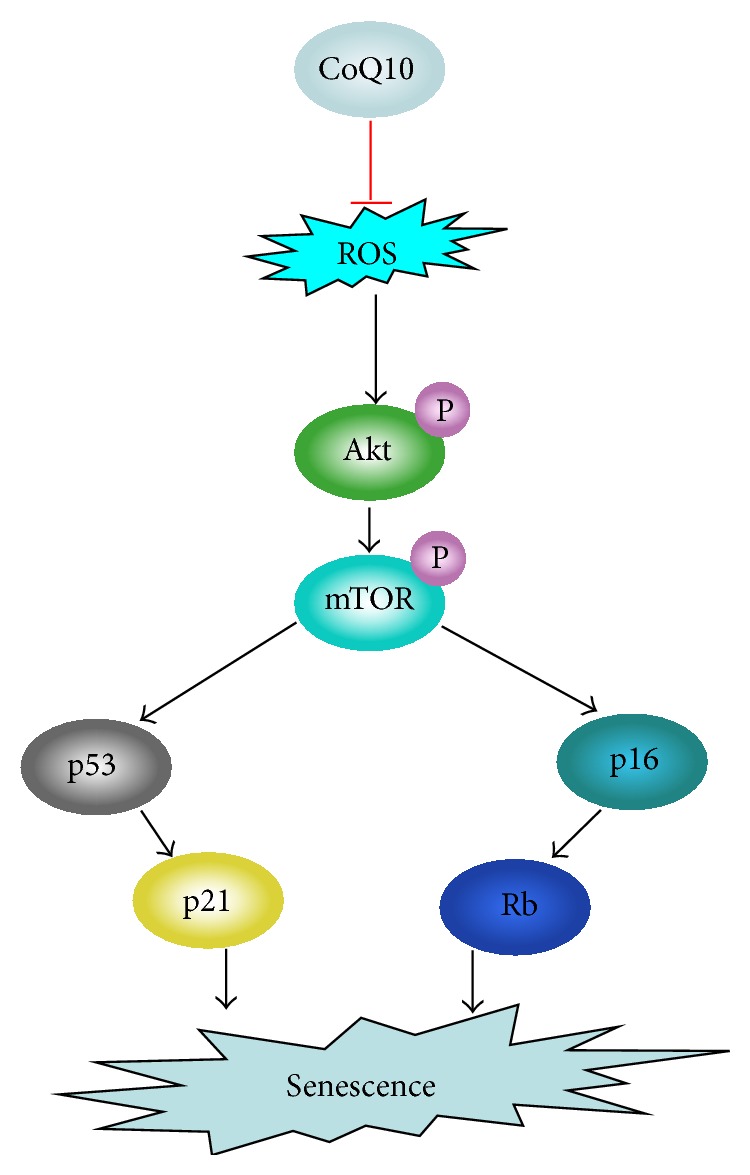
The proposed mechanism of CoQ10 in MSC aging induced by ROS.

In conclusion, D-gal can induce the MSC aging through promoting ROS generation. CoQ10 can obviously decrease the number of SA-*β*-gal-positive cells and the expression of p53, p21, and p16 in D-gal-treated MSCs. CoQ10 may be an effective protection agent of stem cell aging induced by ROS. The Akt/mTOR signaling may be the main mediator of CoQ10 inhibiting MSC senescence (Figure 7). Further deciphering the mechanisms of CoQ10 involved in stem cell aging will help extend the clinical applications and improve the clinical value of CoQ10.
